# Transcriptional alterations of protein coding and noncoding RNAs in triple negative breast cancer in response to DNA methyltransferases inhibition

**DOI:** 10.1186/s12935-021-02213-2

**Published:** 2021-09-26

**Authors:** Ramesh Elango, Radhakrishnan Vishnubalaji, Hibah Shaath, Nehad M. Alajez

**Affiliations:** 1 Translational Cancer and Immunity Center (TCIC), Cancer Research Center, Qatar Biomedical Research Institute (QBRI), Hamad Bin Khalifa University (HBKU), Qatar Foundation (QF), PO Box 34110, Doha, Qatar; 2 College of Health & Life Sciences, Qatar Foundation (QF), Hamad Bin Khalifa University (HBKU), Doha, Qatar

**Keywords:** TNBC, Decitabine, 5-Azacytidine, DNA methyltransferase, lncRNA, TPT1-AS1, MALAT1

## Abstract

**Background:**

DNA methylation plays a crucial role in multiple cellular processes such as gene regulation, chromatin stability, and genetic imprinting. In mammals, DNA methylation is achieved by DNA methyltransferases (DNMTs). A number of studies have associated alterations in DNMT activity to tumorigenesis; however, the exact role of DNMTs in shaping the genome in triple negative breast cancer (TNBC) is still being unraveled.

**Methods:**

In the current study, we employed two DNMT inhibitors (Decitabine and 5-Azacytidine), two TNBC models (MDA-MB-231 and BT-549) and whole transcriptome RNA-Seq and characterized the transcriptional alterations associated with DNMT inhibition. Colony forming unit (CFU), flow cytometry, and fluorescent microscopy were used to assess cell proliferation, cell cycle distribution, and cell death, respectively. Ingenuity pathway analysis (IPA) was used for network and pathway analyses.

**Results:**

Remarkably, DNMT inhibition induced the expression of genes involved in endoplasmic reticulum response to stress, response to unfolder protein, as well as cobalamin metabolic processes. In contrast, suppression of cellular processes related to cell cycle and mitosis were hallmarks of DNMT inhibition. Concordantly, DNMT inhibition led to significant inhibition of TNBC cell proliferation, G2-M cell cycle arrest and induction of cell death. Mechanistically, DNMT inhibition activated TP53, NUPR1, and NFkB (complex) networks, while RARA, RABL6, ESR1, FOXM1, and ERBB2 networks were suppressed. Our data also identified the long noncoding RNA (lncRNA) transcriptional portrait associated with DNMT inhibition and identified 25 commonly upregulated and 60 commonly downregulated lncRNAs in response to Decitabine and 5-Azacytidinec treatment in both TNBC models. TPT1-AS1 was the most highly induced (6.3 FC), while MALAT1 was the most highly suppressed (− 7.0 FC) lncRNA in response to DNMT inhibition.

**Conclusions:**

Taken together, our data provides a comprehensive view of transcriptome alterations in the coding and noncoding transcriptome in TNBC in response to DNMT inhibition.

**Supplementary Information:**

The online version contains supplementary material available at 10.1186/s12935-021-02213-2.

## Background

Breast cancer (BC) remains one of the most common malignant cancers, and the leading cause of cancer-related mortalities among women worldwide [1]. Triple-negative breast cancer (TNBC) has shown to particularly affect women younger in age and tumors are inclined to be larger with higher metastasis, chemo-resistance, relapse frequencies, worse prognosis and relatively poor outcomes in patients [2, 3]. The intra-tumor heterogeneity (ITH) is highly associated with tumorigeneses and untreated tumors are driven to drug-resistance due to genetic and epigenetic modifications [4, 5]. The emergence of resistance in TNBC is an imperative epigenetic challenge to address for the development of better and more effective treatment modalities for TNBC.

Epigenetic mechanisms, which include DNA methylation, posttranslational modification of histone proteins, and gene repression through noncoding RNA (ncRNA) play vital roles under normal physiological and pathological conditions. DNA modification plays an important role in malignant cellular transformation, genomic imprinting, X-chromosome inactivation, gene expression, genetic instability, and mutations, which have been associated with several diseases including cancer [6, 7]. Beyond genetic background, DNA methylation typically occurs at cytosines in the sequence of CpG dinucleotides, which are distributed randomly across the genome. Localized CpG-rich regions, known as CpG islands (CGIs) determine whether genomic regions are transcriptionally active or silent, where highly methylated DNA is associated with transcriptionally inactive genomic regions. Regions rich in GC pairs, such as in CpG islands, are usually unmethylated, serving as a method of gene expression control [8, 9]. The rest of the genome maintains areas of sparse DNA methylated, excluding areas of active transcription sites of genes. Certain CpGs are involved in silencing, genomic imprinting and transcription from repetitive elements, including retroviral genes [10]. Abnormal epigenetic alterations arise in many cancers, regulating the expression patterns of specific genes. Epigenetic dysregulation frequently leads to inappropriate activation or inhibition of multiple signaling pathways and silencing of non-mutated tumor suppressor genes leading to loss of gene function [9, 11, 12].

Recent studies focus on pioneering approaches in treatment of numerous cancers by either inhibiting DNA hypermethylation and/or re-expressing silenced tumor-suppressor genes (TSGs). TSGs usually suppress or negatively regulate cellular proliferation and results in inhibition of tumorigenesis [13]. DNA hypermethylation is mediated through DNA methyl transferases (DNMTs), which can directly silence TSGs expression [14]. CGI hypermethylation in TSGs promoters is a hallmark for cancer. Transcriptional gene silencing and inhibition of transcriptional factors including AP-2, c-Myc/Myn, E2F, and NF-κB, in addition to the recruitment of methyl-CpG binding proteins, has been reported in human cancers including breast cancer [15, 16]. Several genes are found to be hypermethylation in various cancers. It is reported that susceptible genes are involved in cell cycle regulation (Rb), apoptosis, genes associated with DNA repair (BRCA1), transcriptional regulation (hMLH1, Plk2) [17, 18], drug resistance and metastasis [19], thereby preventing hypermethylation by DNA methyl transferase inhibitors (DNMTi’s) [20] represent potential therapeutic intervention for TNBC [20].

Despite the growing advances in epigenetic medicine, there are still numerous challenges in the clinical management of TNBC. The clinically relevant and well characterized DNMT inhibitors, Decitabine and 5-Azacytidine are nucleoside analogue mechanism-based inhibitors, approved by USA Food and Drug Administration (FDA) to treat myelodysplastic syndrome and leukemia [21, 22]. The two aforementioned DNMTi’s may potentially reverse epigenetic alterations resulting in inhibiting cellular proliferation and reactivation of the expression of silenced cancer genes with hypermethylation as shown in preclinical studies for various solid tumors [23, 24]. Data from a randomized clinical phase II study suggested that lower doses of decitabine proved more bearable in ovarian cancer [25]**.** However, clinical trials at present have not been fruitful in solid tumors [26].

In the present study, we employed RNA-seq data geared towards the discovery of the coding and lncRNA transcriptional landscape of TNBC cells treated with DNMTi’s revealing a number of altered biological processes, and the activation of a number of mechanistic networks including TP53, NUPRI and NFkB, while RARA, RABL6, ESR1, FOXM1, and ERBB2 networks were mostly suppressed. We further identified the most highly induced lncRNA, TPT1-AS1, while MALAT1 was more suppressed in response to DNMT inhibition of TNBC models. Our data provides the first transcriptome and network analyses of TNBC cells in repose to DNMTi’s for better understanding of the consequences of DNMT inhibition and their potential utilization for the clinical management of TNBC patients.

## Materials and methods

### Drug preparation

Decitabine and 5-Azacytidine small molecule inhibitors were purchased from Selleckchem (Houston, TX, USA). Inhibitors were dissolved in dimethyl sulfoxide (DMSO) (Sigma Aldrich, St.Louis, MO, USA) at a stock concentration of 10 mM and were stored in aliquots at − 20 °C. Further dilutions were made in DMEM at the time of experiment to achieve final concentrations 2.0 μM.

### Maintenance of TNBC cancer cell lines

Human TNBC (MDA-MB-231 and BT-549) cell lines were cultured in Dulbecco's Modified Eagle Medium (DMEM) supplemented with 10% fetal bovine serum (FBS) and 1% penicillin/streptomycin (Pen-Strep), all were purchased from Thermo Scientific (Thermo Scientific, Rockford, IL, USA). Cells were cultured as an adherent monolayer at 37 °C under 5% CO2 in a humidified incubator.

### RNA isolation and quantification

Forty-eight hours post inhibitor treatment (2.0 μM), total RNA was isolated from treated and control TNBC cells using total RNA purification kit (Norgen Biotek Corp, ON, Canada) as per the manufacturer’s instructions. The concentration and purity of extracted RNA was measured using NanoDrop 2000 (Thermo Scientific, DE, USA) and RNA were stored at − 80 °C.

### Quality assessment of RNA

The quality and quantity of extracted RNA was measured using on-chip electrophoresis utilizing the Agilent RNA 6000 Nano Kit (Agilent Technologies, CA, USA) and Agilent 2100 Bioanalyzer (Agilent Technologies) as per the manufacturer’s instructions. Samples exhibiting an RNA Integrity Number (RIN) > 9 were used for library preparation.

### Total RNA library preparation and RNA sequencing

Total RNA samples with a RIN higher than 9 were used as input for the library preparation using TruSeq Stranded Total RNA Library Prep Gold kit (Cat #: 20020598) from Illumina following the manufacturer’s protocol. Briefly, 500 ng of total RNA was subjected to rRNA depletion and then to fragmentation. The first-strand cDNA synthesis was performed with random hexamers and SuperScript II Reverse Transcriptase (Cat#: 18064014) from ThermoFisher Scientific. The second cDNA strand synthesis was performed by substitution of dTTP with dUTP. The double-stranded cDNA is then end-repaired and adenylated. Barcoded DNA adapters were ligated to both ends of the double-stranded cDNA and then amplified. The libraries quality was checked on an Agilent 2100 Bioanalyzer system and quantified on a Qubit system. The libraries were pooled, clustered on a cBot platform, and sequenced on an Illumina HiSeq 4000 at a minimum of 50 million paired end reads (2 × 75 bp) per sample.

### RNA-Seq and bioinformatics analysis

Pair-end reads were subsequently pseudo aligned to the Gencode release 33 index and reads were subsequently counted using KALLISTO 0.42.1 [27] as we described before [28, 29]. TPM (Transcripts Per Million) expression values were subsequently subjected to differential analysis and hierarchical clustering and Principle component analysis as described before [30]. Transcripts exhibiting − 2.0 ≥ FC ≥ 2.0 and p < 0.05 were considered significant and were used for IPA analysis.

### Ingenuity pathways analysis (IPA)

Differentially expressed genes from the RNA-seq analysis (2.0 FC, p < 0.05) were imported into the IPA Software (Ingenuity Systems Inc., USA) as we previously described [31]. Functional regulatory networks and canonical pathways were determined using upstream regulator analysis (URA), downstream effects analysis (DEA), mechanistic networks (MN), and casual network analysis (CNA) prediction algorithms. Disease and function analysis were used to identify the disease and functional categories affected by DNMTi based on alteration in transcriptome data. IPA uses precise database to paradigm functional regulatory networks from a list of individual genes and determines a statistical score, the Z-score, for each network, according to the fit of the network to the set of focus genes. The biological functions assigned to each network are ranked according to the significance of that biological function to the network [32].

### Cell cycle analysis using flow cytometry

Cell cycle analysis was performed with and without DNMT inhibitors (Decitabine (S1200) and 5-Azacytidine (S1782); Selleckchem) treatments as described before [33]. Briefly, MDA-MB-231 and BT-549 were treated with DNMT inhibitors at 2.0-μM final concentration in 6-well flat-bottom tissue culture plate. On day 3, cells were collected and fixed with 70% ice-cold ethanol and stored at 4° for overnight. Before staining, cells were washed with PBS twice and incubated in RNAse A (100 ug/ml) and propidium iodide (PI; 50 ug/ml) staining solution and then subjected to cell cycle analysis using BD LSRFortessa X-20 flow cytometer (BD Biosciences, CA, USA) at FL3 channel.

### Detection of apoptosis using fluorescence microscopy

The acridine orange and ethidium bromide (AO/EB) fluorescence staining method was used to assess apoptosis in MDA-MB-231 and BT-549 cells after treatment with 2.0 μM of DNMT inhibitors. On day 5, cells were washed and stained with dual fluorescent staining solution containing 100 μg/ml AO and 100 μg/ml EB (AO/EB, Sigma Aldrich, St. Louis, MO, USA) for two minutes; subsequently, imaged under Olympus IX73 fluorescence microscope (Olympus, Tokyo, Japan). The distinction uptake of AO/EB allows the identification of viable and non-viable cells. Principally, AO was used to visualize the number of cells undergone apoptosis, while EB positive cells indicated necrotic cells as we defined before [34].

### Dot blot analysis of DNA cytosine methylation

DNA was isolated using RNA/DNA/Protein Purification plus Kit (Norgen Biotek Corp., ON, Canada) according to manufacturer’s instructions. Briefly, DNA samples was denatured with 0.1 M NaOH at 99 °C for 5 min, neutralized with ammonium acetate, NH4OAc (6 M) as previously described [35]. Different concentration of DNA (20 to 100 ng) in 5 µL blotted onto nitrocellulose membranes (GE Healthcare, Life Science, Germany). The DNA spots was dried at 60 °C for 10 min and further UV-cross linked (60 s, 1200 J/cm2), followed by blocked with 5% non-fat dry milk in tris-buffer saline (TBS) at room temperature for one hour. Subsequently, the level of DNA methylation was analyzed using anti- 5-methylcytosine (5-mC) mouse monoclonal antibody at 1:1000 dilution (Catalogue No: 39649, Active Motif, CA, USA) at overnight at 4 °C incubation. Horseradish peroxidase (HRP)-conjugated rabbit anti-mouse was used as the secondary antibody at 1:2000 dilution. Chemiluminescent detection was performed using WesternSure Chemiluminescent Substrate (LI-COR, Lincoln, NE, USA). Intensities were quantified using the quantification tool in Image Laboratory 5.0 software (Bio-Rad Laboratories).

### TPX2-siRNA transfection and colony-formation in TNBC cells

To investigate the functional consequences of TPX2 knockdown on MDA-MB-231 and BT-549 viability, 0.084 × 10^6^ cells/mL were transfected with the TPX2-siRNAs or scrambled negative control purchased from Ambion. Transfection was performed using a reverse transfection protocol as previously described [33]. In brief, siRNAs at a final concentration of 30 nM were diluted in 50 µL of Opti-MEM (cat no. 11058–021; Gibco, Carlsbad, CA, USA), and 1.5 µL of Lipofectamine 2000 (cat. no. 52758; Invitrogen) was diluted in 50 µL Opti- MEM. The diluted siRNAs and Lipofectamine 2000 were mixed and then incubated at ambient temperature for 20 min. One hundred microliters of transfection mixture were added to the 12-well tissue culture plate, and subsequently 300 µL of MDA-MB-231 and BT-549 (0.084 × 10^6^ cells/mL) in transfection medium (Opti-MEM) were added to each well. The colony-forming ability of TNBC cells transfected with TPX2-siRNAs or siRNA-negative control was determined using a clonogenic assay as described before [31]. In brief, on day 7, the plates were washed and then stained with crystal violet and were subsequently scanned, and the number of colonies were observed under inverted microscope, followed by quantified using 10% of SDS. Similarly, CFU analysis was performed after DNMT inhibitors treatment on day 5.

### Quantitative reverse transcription PCR (qRT-PCR)

500 ng of RNA was used for reverse transcription using High Capacity cDNA Reverse Transcription kit (Applied Biosystems, Foster City, CA, USA). Real time PCR was carried out using PowerUp SYBR Green Master Mix (Applied Biosystems) on QuantStudio 7/6 Flex qPCR (Applied Biosystems) using primer pairs listed in Table 1. Relative levels of transcripts were determined using the 2 ^-^^ΔΔCT^ Method relative to GAPDH reference gene.

**Table 1:** SYBR green primer sequences used in current study

No	Names	Forward sequences	Reverse sequences
1	GAPDH	5′-GGAGCGAGATCCCTCCAAAAT-3′	5′-GGCTGTTGTCATACTTCTCATGG-3′
2	TPX2	5′-AGAAGAGGTGCTCTGAAGGC-3′	5′-CCAGCTGAAAAGGTTCCTGAACTA-3′
3	FTH1	5′-CAGAACTACCACCAGGACTCA-3′	5′-AGTCATCACAGTCTGGTTTCTTG-3′
4	BBC3	5′-CCCGAGATGGAGCCCAATTA-3′	5′-GCTGGGAGTCCAGTATGCTA-3′
5	KLF4	5′-GATGCTCACCCCACCTTCTT-3′	5′-AGTGGTAAGGTTTCTCACCTGT-3′
6	CEBPB	5′-AGCGACGAGTACAAGATCCG-3′	5′-AGCTGCTTGAACAAGTTCCG-3′
7	PTP4A3	5′-CCTCCACCCGTCGTGC-3′	5′-GCAATGGTGAGAGCCAGTG-3′
8	PPARA	5′-TGGACTCAACAGTTTGTGGC-3′	5′-TTCCAGAACTATCCTCGCCG-3′
9	BIRC5	5′-CAGACTTGGCCCAGTGTTTC-3′	5′-GTTCCTCTATGGGGTCGTCA-3′
10	KIF23	5′-TGCTTCCTCGTTGTTTGGAC-3′	5′-CTCTGGATCTACTTGTCGTTTGC-3′
11	MAD2L1	5′-CCGAGTTCTTCTCATTCGGC-3′	5′-ACAAGCAAGGTGAGTCCGTA-3′
12	NCAPG	5′-AGTCCACATAGAGAAGAATGATGC-3′	5′-GGTTGCACTTAAGCCTGTTGA-3′
13	PBK	5′-AGGTACTTGGCCACGACTTA-3′	5′-GCCACTCTCAGTCCAGAGTC-3′
14	PLK4	5′-CGGGGAGAAGATCGAGGATTTTA-3′	5′-CCAGTGTGAATGGACTCAGC-3′

### Statistical and survival analysis

Statistical analyses and graphing were performed using GraphPad Prism 8.0 software (GraphPad, San Diego, CA, USA). The Benjamini–Hochberg False Discovery Rate (FDR) method was used for multiple testing corrections. For IPA analyses, a Z score (− 2.0 ≥ Z ≥ 2.0) was considered significant. The log-rank test was used to compare the outcome between expression groups.

## Results

### DNMT inhibitors predominantly target pathways regulating cell cycle and apoptosis

**Fig. 1:**
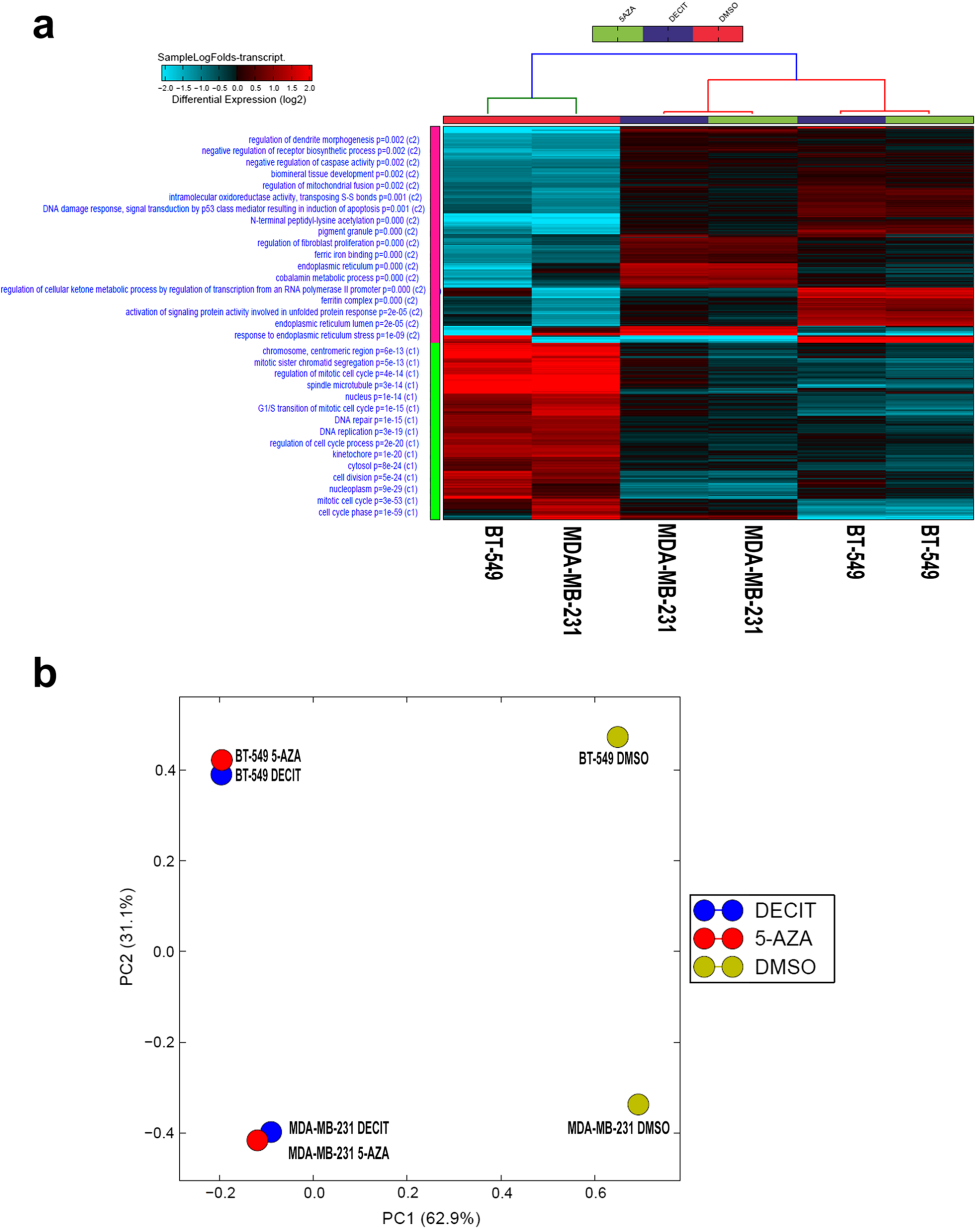
Gene expression analysis of MDA-MB-231 and BT-549 TNBC cells treated with DNMT inhibitors. **a** Hierarchical clustering of MDA-MB-231 and BT-549 TNBC cells treated with Decitabine, 5-Azacytidine, or DMSO control based on differentially expressed genes between the two groups. Each column represents one sample and each row represents a gene. Expression level of each gene (log2) in a single sample is depicted according to the color scale. Enriched functional categories based on gene Ontology (GO) analysis are displayed on the left side. **b** Principal component analysis (PCA) for the RNA transcriptome of MDA-MB-231 and BT-549 TNBC cells treated with Decitabine, 5-Azacytidine, or DMSO control

To characterize the transcriptional landscape alterations in TNBC in response to DNMT inhibition, MDA-MB-231 and BT-549 model were treated with Decitabine and 5-Azacytidine and compared to vehicle-treated controls. RNA was subsequently extracted and was subjected to whole transcriptome RNA-Seq analysis. Decitabine and 5-Azacytidine treatment led to significant inhibition of DNA methylation as shown in Additional file 1. Hierarchical clustering based on differentially expressed mRNA transcripts revealed clear separation of DNMT treated and control group (Fig. 1a). A total of 185 and 227 transcripts were upregulated, while 208 and 149 transcripts were downregulated in response to DNMT inhibition using 5-Azacytidine and Decitabine, respectively (2.0 FC, P < 0.05, Additional file 2: Table S1 and Additional file 3: Table S2). Notably, DNMT inhibition induced the expression of genes involved in response to endoplasmic reticulum stress, response to unfolder protein, cobalamin metabolic processes as well as p53 class mediator induced apoptosis. On the contrary, suppression of cellular processes related to cell cycle and mitosis were the hallmarks of DNMT inhibition. Similar distinction was observed using principal component analysis (PCA; Fig. 1b).

### Downstream effector analysis of differentially expressed genes in TNBC cells treated with DNMT inhibitors

**Fig. 2:**
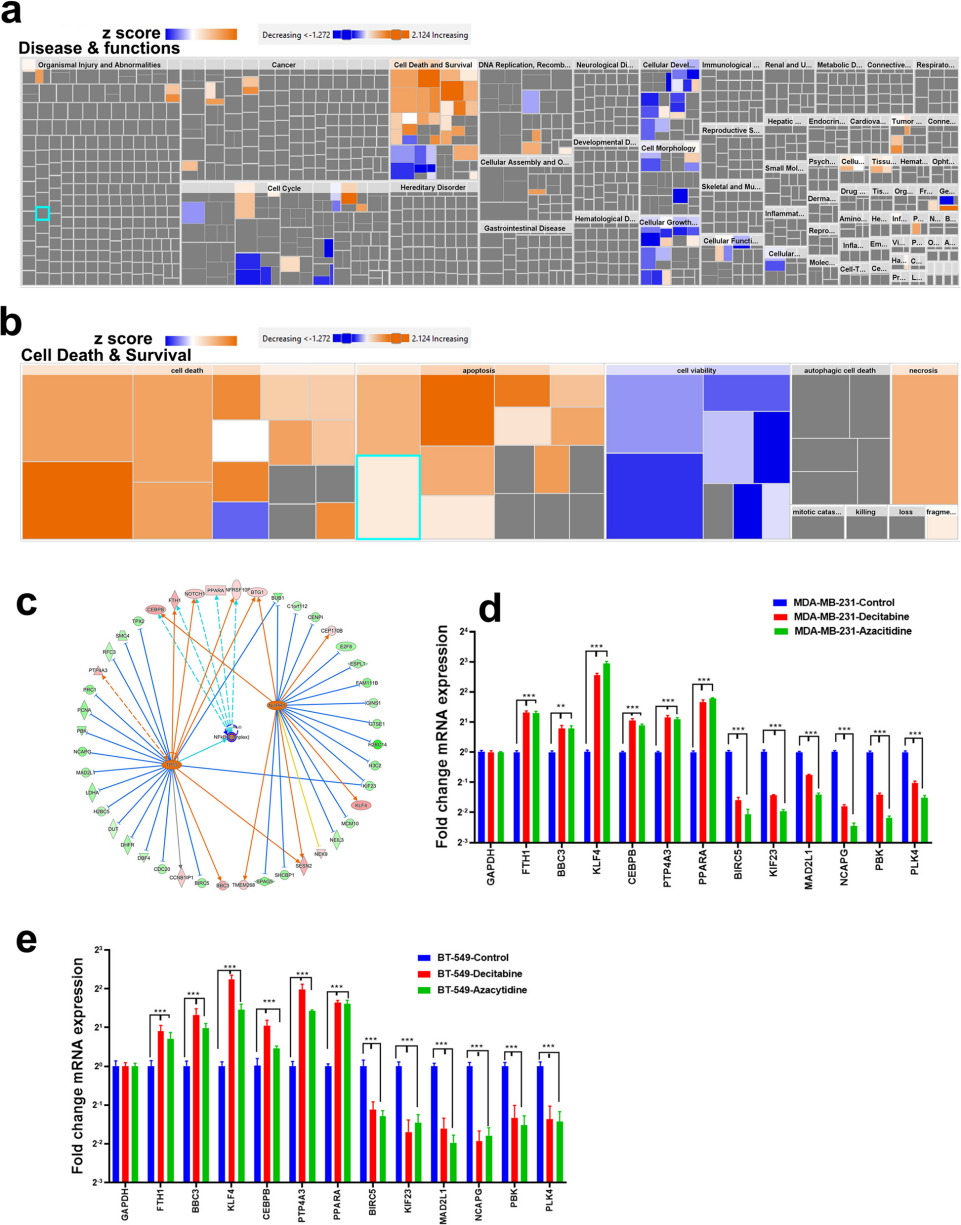
Downstream effector analysis of differentially expressed genes in TNBC cells treated with DNMT inhibitors. **a** Tree map (hierarchical heat map) depicting affected functional categories based on differentially expressed genes in TNBC cells treated with DNMT inhibitors based on RNA-Seq and IPA analysis where the major colored boxes represent a category of diseases and functions where blue indicated decreasing while orange indicate increasing activation Z score. **b** Illustration of cell death and survival functional category. **c** Illustration of NUPR1, TP53, and NFKB mechanistic networks. The expression of selected number of differentially expressed genes was validated using qRT-PCR in MDA-MB-231 **d** and BT-549. **e** Data are presented as mean ± SEM (n = 6). *P < 0.05, **P < 0.005, ***P < 0.0005

We subsequently performed gene set enrichment analysis using the Ingenuity Pathway Analysis (IPA) tool on the differentially expressed transcripts in TNBC cells in response to DNMT inhibition. IPA analysis revealed several altered disease and functional categories in response to DNMT inhibition (Fig. 2a, Additional file 4: Table S3). Notably, cell death functional categories such as apoptosis and necrosis were the most activated in DNMTi treated TNBC cells (Fig. 2b). Upstream regulator analysis revealed three transcriptional regulatory networks (TP53, NUPR1, and NFkB complex) to be significantly activated (activation z score 4.7, 4.2, and 2.2 respectively) in response to DNMTi treatment of TNBC cells (Fig. 2c). Notably, TP53 was predicted to activate eight gene targets (*FTH1, NOTCH1, TNFRSF10B, BTG1, SESN2, BBC3, CCNB1IP1,* and *PTP4A3),* as well as inducing its own activation through a positive feedback loop. An additional 17 gene targets were repressed (*BIRC5, BUB1, CDC20, DBF4, DHFR, DUT, H2BC5, KIF23, LDHA, MAD2L1, NCAPG, PBK, PCNA, PRC1, RFC3, SMC4, and TPX2)*. NUPR1 network activated six gene targets (*CEBPB, BTG1, CEP170B, KLF4, SESN2, and TMEM268*) and 16 gene targets (*BUB1, C1orf112, CENPI, E2F8, ESPL1, FAM111B, GINS1, GTSE1, H2BC14, H3C2, KIF23, MCM10, NEIL3, NEK9, SHCBP1, and SPAG5*) were repressed. NFkB complex is predicted to be associated with five gene targets (*CEBPB, FTH1, NOTCH1, PPARA, and TNFRSF10B*) with activational effect (Fig. 2c, Additional file 5: Table S5). The expression of selected number of differentially expressed genes (FTH1, BBC3, KLF4, CEBPB, PTP4A3, PPARA, BIRC5, KIF23. MAD2L1, NCAPG, PBK, and PLK4) was validated using qRT-PCR in MDA-MB-231 and BT-549 (Fig. 2d, e).Taken together, our data highlighted activation of several tumor suppressor transcriptional regulatory networks in response to DNMT inhibition leading to tumor cell death.

### Repressed mechanistic networks leads to apoptosis in response to DNMT inhibition

**Fig. 3:**
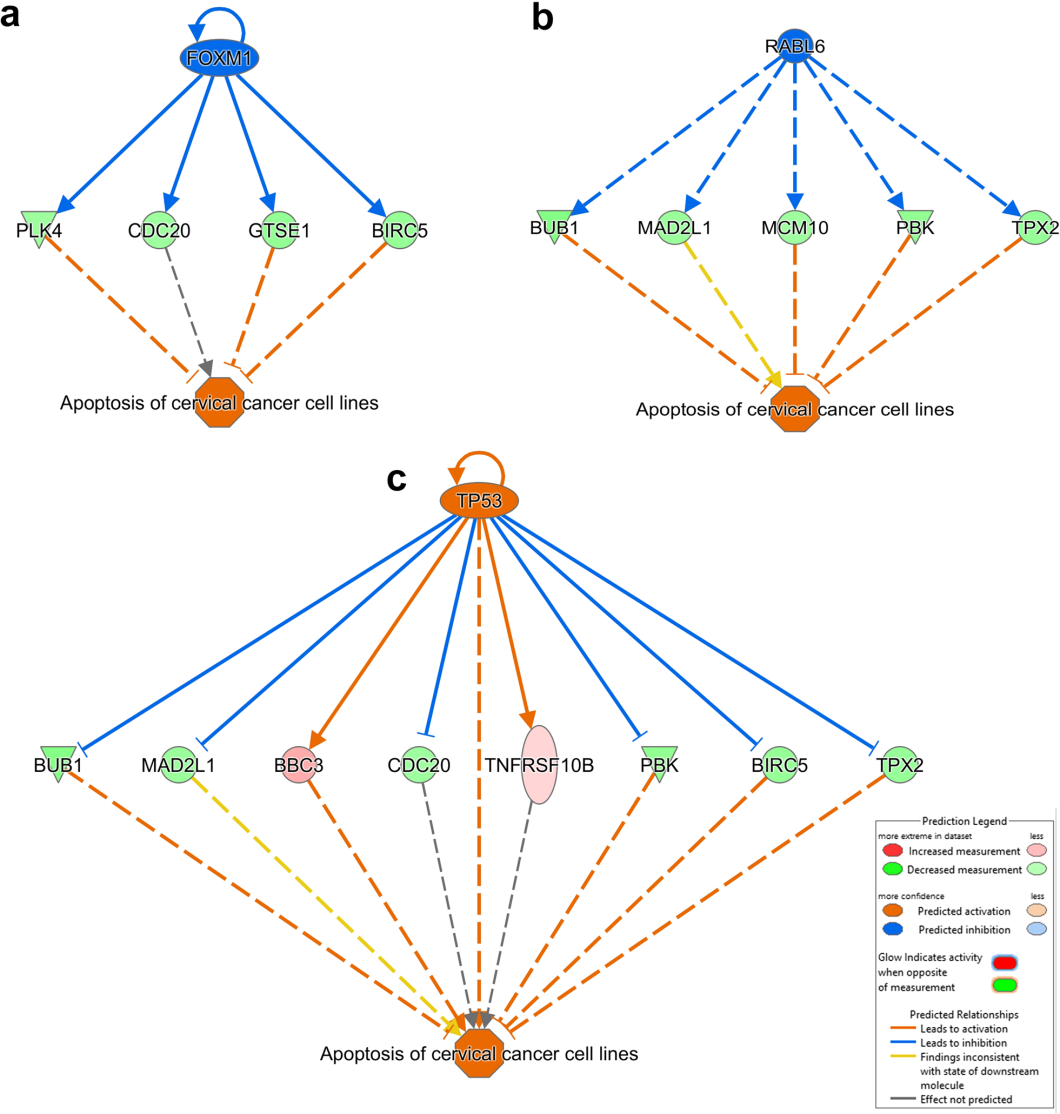
Regulator effects network analysis of TNBC cells treated with DNMT inhibitors. Illustration of inhibited FOXM1 **a** and RABL6 **b** as well as the activated TP53 **c** regulator effects networks with predicted activated state of the network based on transcriptome data with subsequent predicted effects on downstream effector molecules. Legend illustrate the relationship between molecules within the network

Upstream regulator analysis of the differentially expressed genes revealed dramatic effects of DNMT inhibitors on numerous vital networks in TNBC cells. Notably, the most inhibited networks were those driven by CREB1, E2F3, KDM1A, MITF, ERBB2, FOXM1, ESR1, RABL6 and RARA upstream regulators (Additional file 6: Table S5). In particular, FOXM1 and RARA networks were highly suppressed in response to DNMT inhibition, including self-inhibition by FOXM1. The regulator effects of those networks are represented in Fig. 3a, b, with their downstream effects on cellular apoptosis. On the other hand, activated networks include TP53 (Fig. 3c), which also triggers apoptosis in DNMTi treated cells. Taken together, our data highlighted a number of upstream regulator networks affected by DNMT inhibitors, which collectively promotes dell death.

### Functional consequences of DNMT inhibition on TNBC cell viability

**Fig. 4:**
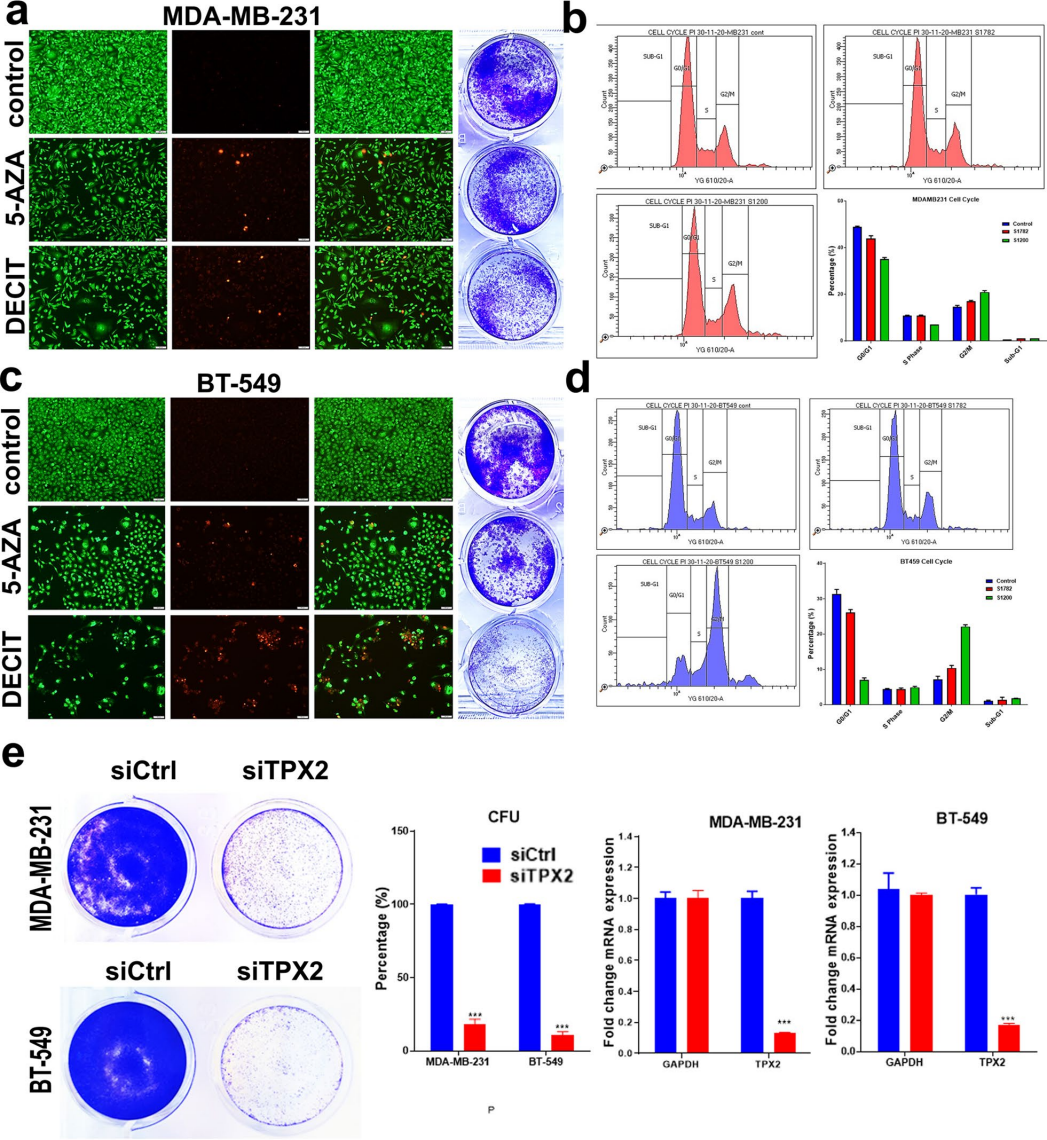
Functional consequences of DNMTi on TNBC cells. MDA-MB-231 (**a**, **b**) and BT-549 (**c**, **d**) cells were treated with Decitabine and 5-Azacytidine at 2.0 µM. Cells were stained with AO/EO to detect apoptotic (cells with green condensed chromatin) and dead (red) cells (**a**, **c**). CFU assay was used to assess colony formation potential of MDA-MB-231 and BT-549 treated with DNMTi. Plates were stained with crystal violet on day 5. Data are representative of two independent experiments for each condition. Cell cycle analysis demonstrating the effects of DNMT inhibitors on cell cycle distribution in TNBC models (**b**, **d**). Quantification of cell cycle distribution is presented as bar diagram (n = 3). **e** CFU and qRT-PCR for TPX2 expression of MDA-MB-231 and BT-549 after treatment with siRNA targeting TPX2 or control siRNA on day 7. Quantification of CFU staining is presented on the right panel

In order to validate the functional effects of DNMT inhibitors in the TNBC models, both MDA-MB-231 and BT-549 cells were treated with Decitabine and 5-Azacytidine and were subjected to AO/EB staining to assess cell death, CFU to assess cell proliferation and cell cycle analysis to assess changes in cell cycle distribution. AO/EB staining revealed significant inhibition of cellular proliferation and induction of apoptosis and necrosis in both models (Fig. 4a, c). The data are concordant with CFU which also revealed substantial inhibition of colony forming capabilities of TNBC cells in response to DNMT inhibition. Cell cycle analysis revealed significant G2-M cell arrest, reduction in G0-G1phase, and an increase in subG0 (apoptotic), which is collectively concordant with IPA analysis (Fig. 4b, d). IPA analysis revealed suppression of TPX2 by TP53 in response to DNMT inhibition (Fig. 2c and Additional File 5). To corroborate those findings, we used siRNA to assess the consequences of TPX2 depletion on TNBC CFU potential. Data presented in Fig. 4e revealed significant reduction in TPX2 expression in siTPX2-transfecetd TNBC cells which led to subnational inhibition of CFU of MDA-MB-231 and BT-549 cells suggesting the TP53-TPX2 axis as potential mechanism by which DNMTi exert their biological function.

### Prognostic value of the gene signatures in TNBC cells treated with DNMT inhibitors

**Fig. 5:**
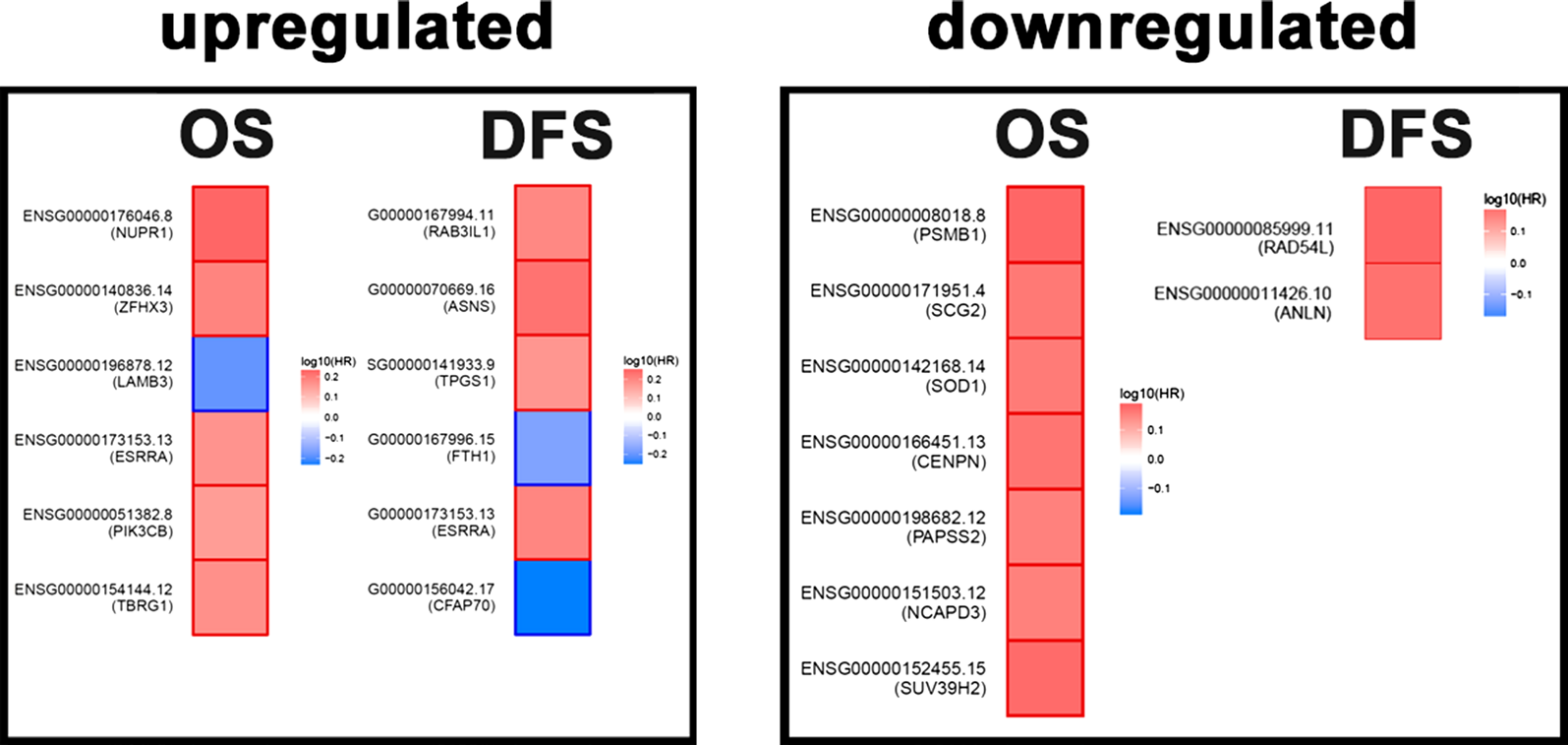
Prognostic value of gene signatures based on differentially expressed genes in TNBC cells treated with DNMT inhibitors. Survival heatmap for the upregulated and downregulated genes for overall (OS) and disease free (DFS) survival based on GEPIA2 database. Red color indicated HR > 1 while blue color indicates HR < 1.Only genes with significant HR (p value < 0.05) are shown

The prognostic value of upregulated and downregulated gene signatures in response to DNMT inhibition in TNBC to overall survival (OS) and disease-free survival (DFS) were evaluated using hazard ratio (HR) in GEPIA2 database (Fig. 5). Squares outlined with darker edges have the highest prognostic values. Interestingly, five upregulated genes (*TBRG1, PIK3CB, ESSRA, ZFHX3, and NUPR1)* were associated with worse OS, while CLIP2 and LAMB3 were associated with better OS. *ESSRA, TPGS1, ASNS,* and *RAB3L1* exhibited worse DFS, while *WIZ, CFAP70,* and *FTH1* were associated with better DFS. Looking into downregulated genes in response to DNMT treatment, *SUV39H2, NCAPD3, PAPSS2, CENPN, SOD1, SCO2,* and *PSMB1* were associated with worse OS, while *ANLN* and *RAD54L* were associated with worse DFS (Fig. 5).

### Expression profiling of lncRNAs in TNBC cells in response to DNMT inhibitors

**Fig. 6:**
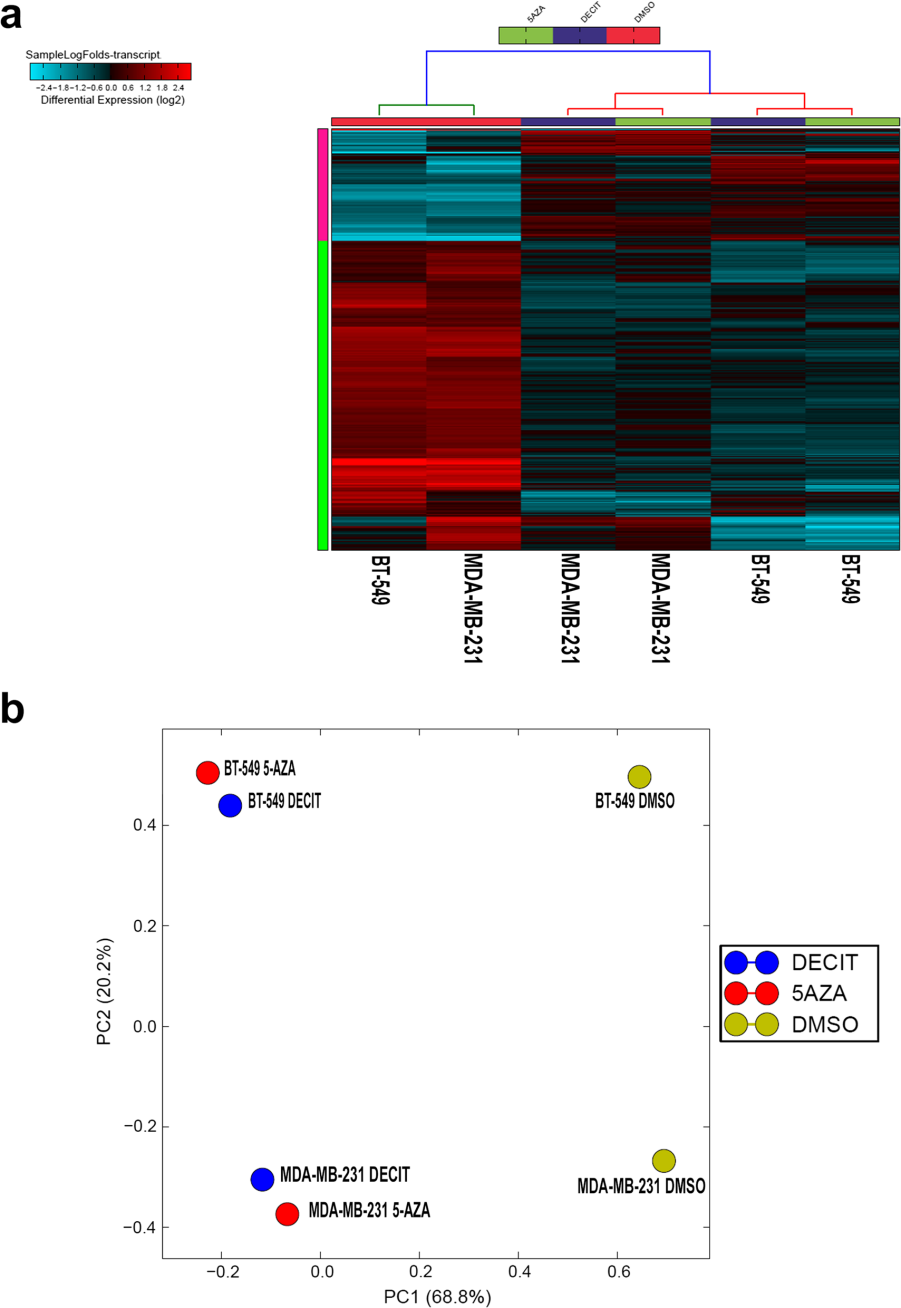
Long non-coding RNA (lncRNA) transcriptome in MDA-MB-231 and BT-549 TNBC cells treated with DNMT inhibitors. **a** Hierarchical clustering of MDA-MB-231 and BT-549 TNBC cells treated with Decitabine, 5-Azacytidine, or DMSO control based on differentially expressed lncRNA transcripts between the two groups. Each column represents one sample and each row represents a transcript. Expression level of each lncRNA (log2) in a single sample is depicted according to the color scale. **b** Principal component analysis (PCA) for MDA-MB-231 and BT-549 TNBC cells treated with Decitabine, 5-Azacytidine, or DMSO control based on lncRNA transcriptome

**Fig. 7:**
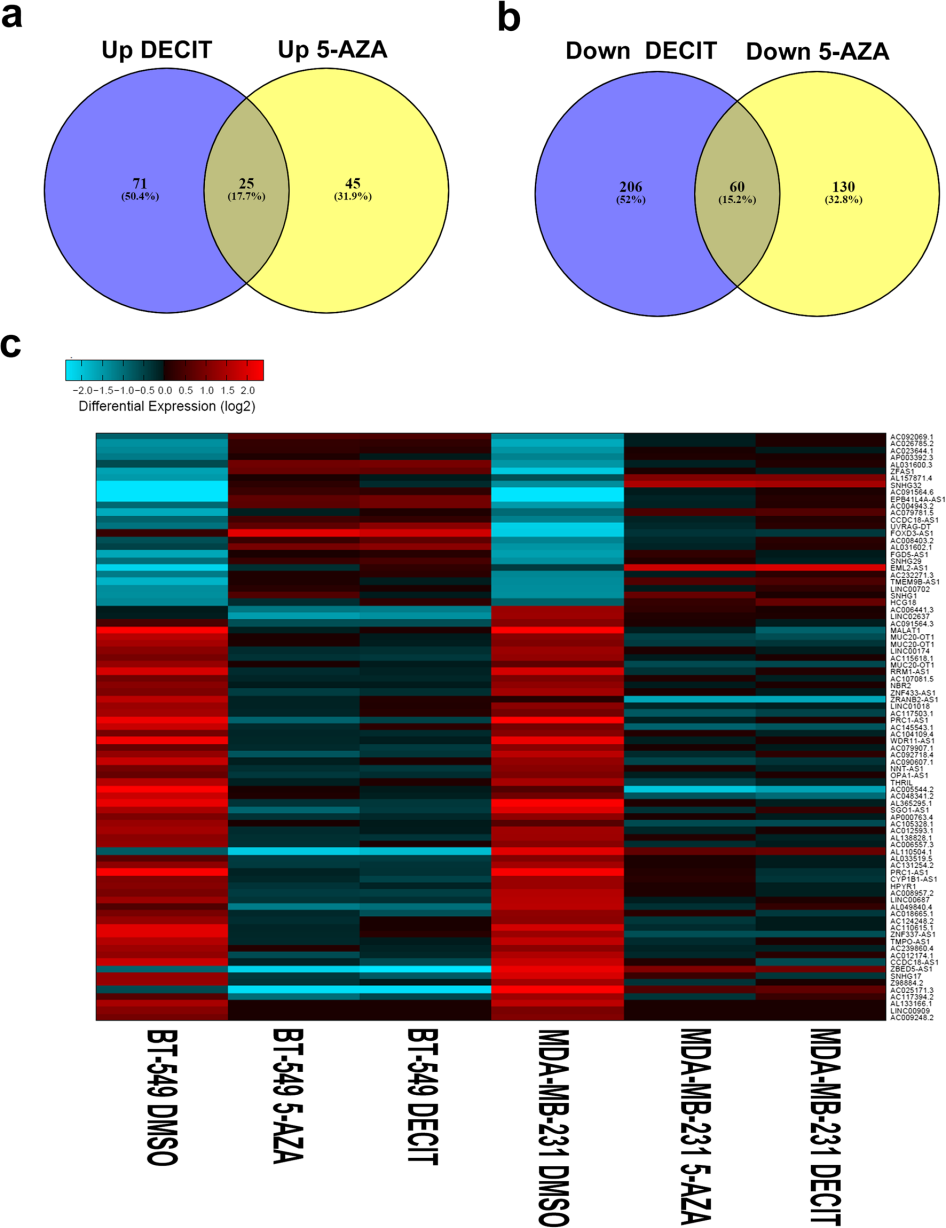
Commonly affected lncRNA transcripts in response to DNMT inhibitor treatment. Venn diagram illustrating commonly upregulated (**a**) or downregulated (**b**) lncRNA transcripts in MDA-MB-231 and BT-549 cells treated with Decitabine or 5-Azacytidine. **c** Heat map depicting the expression of 25 commonly upregulated and 60 commonly downregulated lncRNA transcripts in MDA-MB-231 and BT-549 TNBC cells treated with Decitabine, 5-Azacytidine compared to DMSO control treated cells

To gain more insight into changes in differentially expressed lncRNA transcripts in TNBC cells treated with Decitabine and 5-Azacytidine compared to vehicle-treated control cells, we utilized RNA-Seq data and computational analysis. Transcriptome data were mapped to the Gencode release 33 followed by differential expression analysis to determine the lncRNA transcripts affected by DNMTi treatment. As depicted in Fig. 6a, hierarchical clustering revealed two major clusters, where control samples clustered to the left side, followed by MDA-MB-231 treated with Decitabine and 5-Azacytidine and BT-549 cells treated with Decitabine and 5-Azacytidine. A total of 70 common lncRNA transcripts were upregulated, while 190 common lncRNA transcripts were downregulated in response to 5-Azacytidine, while 97 common lncRNA transcripts were upregulated and 266 common lncRNA transcripts were downregulated in response to Decitabine in the two TNBC models (Additional file 6: Table S5, Additional file 7: Table S6). Principle component analysis (PCA) also revealed clear separation of TNBC cells treated with Decitabine and 5-Azacytidine compared to the vehicle control based on lncRNA transcriptome (Fig. 6b). We subsequently crossed the differentially expressed lncRNA list from 5-Azacytidine and Decitabine and identified 25 commonly upregulated and 60 commonly downregulated lncRNA transcripts in response to the two DNMT inhibitors (Fig. 7a, b; Additional file 8: Table S7). Expression of commonly upregulated and downregulated lncRNAs in response to 5-Azacytidine and Decitabine is presented as heatmap in Fig. 7c. Interestingly, TPT1-AS1 was the most highly induced (6.3 FC), while MALAT1 was the most highly suppressed (− 7.0 FC) lncRNA in response to DNMT inhibition (Additional files 6 and 7). Taken together, our data highlighted the transcriptional alterations in the coding and noncoding transcriptome of TNBC in response to DNMT inhibition.

## Discussion

Our comprehension of epigenetic regulation has rapidly increased dramatically over the past decades. Advanced technologies in gene expression analysis of the coding and untranslated regions of the genome have enabled us to evaluate DNA methylation patterns, facilitating the identification of actionable target pathways in the cancer epigenome [8, 9]. In the current study, we analyzed the transcriptional alterations of two TNBC models in response to Decitabine and 5-Azacytidine treatment. While several of the identified transcripts could be directly regulated by DNA methylation, we do not exclude the possibility that a number of the differentially expressed genes in our experiments could be indirect consequence of DNMT inhibition. Our data supports a multi-pronged effects of DNMT inhibition though induction of genes involved in the response to endoplasmic reticulum stress, response to unfolded protein, cobalamin metabolic processes as well p53-mediaed apoptosis. On the other hand, suppression of cellular processes related to cell cycle and mitosis, which corroborates the earlier findings, reported that genes associated with CpGs and P53 pathways regulates DNA repair and apoptosis in several types of human cancers including lung [36], and colorectal cancers [37].

Mechanistic network analyses revealed activation of manifold networks with NUPR1, TP53, and NFKB signaling on top of the list, while RARA, RABL6, ESR1, FOXM1, and ERBB2 networks were suppressed. Our data is concordant with other studies highlighting an important role for NUPR1 [38], TP53 [39], and NFkB [40] in several cancers, thereby supporting that the activated pathways have promising therapeutic potential for patients with TNBC.

Among the identified mechanistic networks in current study, CREB, E2F3, and KDM1A were previously shown to activate transcriptional programs and to promote cellular growth, migration, cell cycle progression and DNA damage response [44–46]. Another group reported that CREB plays an important role in cellular migration and contributes to the epithelial-to-mesenchymal transition (EMT) of human breast cancer [47]. FOXM1, a transcription factor upregulated in several cancer types, plays a key role in cell cycle progression, stemness and tumorigenesis [48]. Our previous studies and other groups have also highlighted the role of FOXM1 activation in colorectal cancer and TNBC [29, 49]. Overexpression of FOXM1 and ERBB2 lead to genomic instability and uncontrolled cell division and malignancy, which are associated with poor prognosis in various cancerous lesions including breast cancers [50, 51]. Our previous study in colorectal cancer reported FOXM1 to be a novel target for epigenetic regulation by the miR-320 family [52]. Other research groups revealed that small molecule inhibitors, (naphthol AS‐E) mediated CREB gene transcription through inhibiting cell proliferation, migration, and survival in breast cancer cells [53]. Our studies revealed that the most inhibited transcription networks by DNMT inhibitors were CREB1, E2F3, KDM1A, MITF, ERBB2, FOXM1, ESR1, RABL6 and RARA. Our findings from the current study are concordant with our previously published work on transcription factors such as ERBB2, RABL6, FOXM1 and MITF which are most effected by palbociclib treatment in MDA-MB-231 breast cancer cells, reducing colony formation, cell migration and viability [33]. Our current data corroborated a role for TPX2 in regulating TNBC proliferation and colony formation potential. Our data could explain in part the inhibitory effects of DNMTi though downregulation of TPX2 though TP53 activation.

LncRNAs have recently been identified as key epigenetic regulators of multiple biological functions including cellular differentiation, migration and cancer progression through chromatin remodeling, DNA methylation, transcriptional and posttranscriptional regulation [60, 61]. In addition to regulation of protein coding mRNA transcripts, our data revealed regulation of several lncRNAs by DNMTi. Our data identified TPT1-AS1 as the most highly induced lncRNA (6.3 FC) in response to DNMT inhibition in TNBC cells. A number of studies reported oncogenic role for TPT1-AS1 in ovarian and colorectal cancer. However, in breast cancer low expression of TPT1-AS1 was associated with high tumor, nodes, and metastases (TNM) stage, lymph node metastasis and predicted shorter overall survival [72]. Our data revealed epigenetic regulation of TPT1-AS1 by DNA methylation as potential mechanism leading to its suppressed expression in breast cancer. MALAT1 was the most downregulated lncRNA in response to DNMTi of TNBC. Our recent data implicated MALAT1 in TNBC resistance to neoadjuvant chemotherapy where we showed CRISPR-Cas9 mediated MALAT1 promoter deletion to reduce CFU and enhance sensitivity of TNBC cells to chemotherapy, hence corroborating data from current study [73].

## Conclusions

In conclusion, our data provides a comprehensive view of transcriptomic alteration in the coding and noncoding transcriptome of TNBC cells in response to DNMT inhibition. Our data contributes toward our understanding of the mechanism by which DNMT inhibition induce TNBC cell death through widespread regulation of the genome and suggest their therapeutic potential to treat patients with TNBC.

## Supplementary Information

**Additional file 1: Figure S1.** 5-mC-specific dot blot assay of gDNA of DNMT inhibitors treated TNBC models.

**Additional file 2: Table S1.** Differentially expressed genes in MDA-MB-231 and BT-549 treated with 5-Azacytidine.

**Additional file 3: Table S2.** Differentially expressed genes in MDA-MB-231 and BT-549 treated with Decitabine.

**Additional file 4: Table S3.** Affected functional categories in MDA-MB-231 and BT-549 TNBC cells treated with Decitabine, 5-Azacytidine compared to DMSO control treated cells based on RNAseq and IPA analysis.

**Additional file 5: Tables S4.** Affected upstream regulator networks in MDA-MB-231 and BT-549 TNBC cells treated with Decitabine, 5-Azacytidine compared to DMSO control treated cells.

**Additional file 6: Table S5.** Differentially expressed lncRNAs in MDA-MB-231 and BT-549 treated with 5-Azacytidine.

**Additional file 7: Table S6.** Differentially expressed lncRNAs in MDA-MB-231 and BT-549 treated with Decitabine.

**Additional file 8: Table S7.** Common upregulated and downregulated lncRNA transcripts in lncRNA transcripts in MDA-MB-231 and BT-549 TNBC cells treated with Decitabine, 5-Azacytidine compared to DMSO control treated cells.

## Data Availability

All data are fully available without restrictions.

## Abbreviations

DNMTs: DNA methyltransferases
DNMTi’s: DNA methyl transferase inhibitors
TNBC: Triple negative breast cancer
lncRNAs: Long non-coding RNAs
ncRNA: Noncoding RNA
ITH: Intra-tumor heterogeneity
TSGs: Tumor-suppressor genes
EMT: Epithelial-to-mesenchymal transition
DMEM: Dulbecco's Modified Eagle Medium
FBS: Fetal bovine serum
IPA: Ingenuity pathways analysis
URA: Upstream regulator analysis
DEA: Downstream effects analysis
OS: Overall survival
DFS: Disease free survival
TNM: Tumor, nodes, and metastases

## References

[1] McGuire A, Brown JA, Malone C, McLaughlin R, Kerin MJ (2015). Effects of age on the detection and management of breast cancer. Cancers (Basel).

[2] Dent R, Trudeau M, Pritchard KI, Hanna WM, Kahn HK, Sawka CA, Lickley LA, Rawlinson E, Sun P, Narod SA (2007). Triple-negative breast cancer: clinical features and patterns of recurrence. Clin Cancer Res.

[3] Perou CM, Sorlie T, Eisen MB, van de Rijn M, Jeffrey SS, Rees CA, Pollack JR, Ross DT, Johnsen H, Akslen LA (2000). Molecular portraits of human breast tumours. Nature.

[4] Dagogo-Jack I, Shaw AT (2018). Tumour heterogeneity and resistance to cancer therapies. Nat Rev Clin Oncol.

[5] Salgia R, Kulkarni P (2018). The genetic/non-genetic duality of drug 'resistance' in cancer. Trends Cancer.

[6] Greenberg MVC, Bourc'his D (2019). The diverse roles of DNA methylation in mammalian development and disease. Nat Rev Mol Cell Biol.

[7] Ahuja N, Sharma AR, Baylin SB (2016). Epigenetic therapeutics: a new weapon in the war against cancer. Annu Rev Med.

[8] Baylin SB, Jones PA (2011). A decade of exploring the cancer epigenome—biological and translational implications. Nat Rev Cancer.

[9] Shen H, Laird PW (2013). Interplay between the cancer genome and epigenome. Cell.

[10] Bestor TH, Edwards JR, Boulard M (2015). Notes on the role of dynamic DNA methylation in mammalian development. Proc Natl Acad Sci USA.

[11] Jones PA, Issa JP, Baylin S (2016). Targeting the cancer epigenome for therapy. Nat Rev Genet.

[12] Garraway LA, Lander ES (2013). Lessons from the cancer genome. Cell.

[13] Jones PA, Laird PW (1999). Cancer epigenetics comes of age. Nat Genet.

[14] Rosic S, Amouroux R, Requena CE, Gomes A, Emperle M, Beltran T, Rane JK, Linnett S, Selkirk ME, Schiffer PH (2018). Evolutionary analysis indicates that DNA alkylation damage is a byproduct of cytosine DNA methyltransferase activity. Nat Genet.

[15] Zhu H, Wang G, Qian J (2016). Transcription factors as readers and effectors of DNA methylation. Nat Rev Genet.

[16] Yin Y, Morgunova E, Jolma A, Kaasinen E, Sahu B, Khund-Sayeed S, Das PK, Kivioja T, Dave K, Zhong F (2017). Impact of cytosine methylation on DNA binding specificities of human transcription factors. Science.

[17] Watanabe Y, Ueda H, Etoh T, Koike E, Fujinami N, Mitsuhashi A, Hoshiai H (2007). A change in promoter methylation of hMLH1 is a cause of acquired resistance to platinum-based chemotherapy in epithelial ovarian cancer. Anticancer Res.

[18] Syed N, Coley HM, Sehouli J, Koensgen D, Mustea A, Szlosarek P, McNeish I, Blagden SP, Schmid P, Lovell DP (2011). Polo-like kinase Plk2 is an epigenetic determinant of chemosensitivity and clinical outcomes in ovarian cancer. Cancer Res.

[19] Koch A, Joosten SC, Feng Z, de Ruijter TC, Draht MX, Melotte V, Smits KM, Veeck J, Herman JG, Van Neste L (2018). Analysis of DNA methylation in cancer: location revisited. Nat Rev Clin Oncol.

[20] Abbotts R, Topper MJ, Biondi C, Fontaine D, Goswami R, Stojanovic L, Choi EY, McLaughlin L, Kogan AA, Xia L (2019). DNA methyltransferase inhibitors induce a BRCAness phenotype that sensitizes NSCLC to PARP inhibitor and ionizing radiation. Proc Natl Acad Sci USA.

[21] Kaminskas E, Farrell A, Abraham S, Baird A, Hsieh LS, Lee SL, Leighton JK, Patel H, Rahman A, Sridhara R (2005). Approval summary: azacitidine for treatment of myelodysplastic syndrome subtypes. Clin Cancer Res.

[22] Peterlin P, Cluzeau T, Jullien M, Ngo Nloga AM, Calleja A, Angeli E, Chevallier P, Guillaume T, Garnier A, Le Bourgeois A (2020). Azacitidine in patients older than 80 years with acute myeloid leukaemia or myelodysplastic syndromes: a report on 115 patients. Br J Haematol.

[23] Morel D, Jeffery D, Aspeslagh S, Almouzni G, Postel-Vinay S (2020). Combining epigenetic drugs with other therapies for solid tumours—past lessons and future promise. Nat Rev Clin Oncol.

[24] Silverman LR, Fenaux P, Mufti GJ, Santini V, Hellstrom-Lindberg E, Gattermann N, Sanz G, List AF, Gore SD, Seymour JF (2011). Continued azacitidine therapy beyond time of first response improves quality of response in patients with higher-risk myelodysplastic syndromes. Cancer.

[25] Glasspool RM, Brown R, Gore ME, Rustin GJ, McNeish IA, Wilson RH, Pledge S, Paul J, Mackean M, Hall GD (2014). A randomised, phase II trial of the DNA-hypomethylating agent 5-aza-2'-deoxycytidine (decitabine) in combination with carboplatin vs carboplatin alone in patients with recurrent, partially platinum-sensitive ovarian cancer. Br J Cancer.

[26] Appleton K, Mackay HJ, Judson I, Plumb JA, McCormick C, Strathdee G, Lee C, Barrett S, Reade S, Jadayel D (2007). Phase I and pharmacodynamic trial of the DNA methyltransferase inhibitor decitabine and carboplatin in solid tumors. J Clin Oncol.

[27] Bray NL, Pimentel H, Melsted P, Pachter L (2016). Near-optimal probabilistic RNA-seq quantification. Nat Biotechnol.

[28] Elango R, Alsaleh KA, Vishnubalaji R, Manikandan M, Ali AM, Abd El-Aziz N, Altheyab A, Al-Rikabi A, Alfayez M, Aldahmash A (2020). MicroRNA expression profiling on paired primary and lymph node metastatic breast cancer revealed distinct microRNA profile associated with LNM. Front Oncol.

[29] Shaath H, Toor SM, Nair VS, Elkord E, Alajez NM (2019). Transcriptomic analyses revealed systemic alterations in gene expression in circulation and tumor microenvironment of colorectal cancer patients. Cancers (Basel).

[30] Emig D, Salomonis N, Baumbach J, Lengauer T, Conklin BR, Albrecht M (2010). AltAnalyze and DomainGraph: analyzing and visualizing exon expression data. Nucleic Acids Res.

[31] Vishnubalaji R, Elango R, Al-Toub M, Manikandan M, Al-Rikabi A, Harkness L, Ditzel N, Atteya M, Hamam R, Alfayez M (2019). Neoplastic transformation of human mesenchymal stromal cells mediated via LIN28B. Sci Rep.

[32] Kramer A, Green J, Pollard J, Tugendreich S (2014). Causal analysis approaches in ingenuity pathway analysis. Bioinformatics.

[33] Elango R, Vishnubalaji R, Manikandan M, Binhamdan SI, Siyal AA, Alshawakir YA, Alfayez M, Aldahmash A, Alajez NM (2019). Concurrent targeting of BMI1 and CDK4/6 abrogates tumor growth in vitro and in vivo. Sci Rep.

[34] Elango R, Vishnubalaji R, Shaath H, Alajez NM (2021). Molecular subtyping and functional validation of TTK, TPX2, UBE2C, and LRP8 in sensitivity of TNBC to paclitaxel. Mol Ther Methods Clin Dev.

[35] Greco CM, Kunderfranco P, Rubino M, Larcher V, Carullo P, Anselmo A, Kurz K, Carell T, Angius A, Latronico MV (2016). DNA hydroxymethylation controls cardiomyocyte gene expression in development and hypertrophy. Nat Commun.

[36] Kalbe B, Schulz VM, Schlimm M, Philippou S, Jovancevic N, Jansen F, Scholz P, Lubbert H, Jarocki M, Faissner A (2017). Helional-induced activation of human olfactory receptor 2J3 promotes apoptosis and inhibits proliferation in a non-small-cell lung cancer cell line. Eur J Cell Biol.

[37] Li XL, Zhou J, Chen ZR, Chng WJ (2015). P53 mutations in colorectal cancer-molecular pathogenesis and pharmacological reactivation. World J Gastroenterol.

[38] Emma MR, Iovanna JL, Bachvarov D, Puleio R, Loria GR, Augello G, Candido S, Libra M, Gulino A, Cancila V (2016). NUPR1, a new target in liver cancer: implication in controlling cell growth, migration, invasion and sorafenib resistance. Cell Death Dis.

[39] Mukhopadhyay UK, Oturkar CC, Adams C, Wickramasekera N, Bansal S, Medisetty R, Miller A, Swetzig WM, Silwal-Pandit L, Borresen-Dale AL (2019). TP53 status as a determinant of pro- vs anti-tumorigenic effects of estrogen receptor-beta in breast cancer. J Natl Cancer Inst.

[40] Lehman HL, Kidacki M, Warrick JI, Stairs DB (2018). NFkB hyperactivation causes invasion of esophageal squamous cell carcinoma with EGFR overexpression and p120-catenin down-regulation. Oncotarget.

[41] Badis G, Berger MF, Philippakis AA, Talukder S, Gehrke AR, Jaeger SA, Chan ET, Metzler G, Vedenko A, Chen X (2009). Diversity and complexity in DNA recognition by transcription factors. Science.

[42] Huang T, Wang G, Yang L, Peng B, Wen Y, Ding G, Wang Z (2017). Transcription factor YY1 modulates lung cancer progression by activating lncRNA-PVT1. DNA Cell Biol.

[43] Testa AC, Forrest ARR (2016). Transcription factor NKX6.3 sheds light on gastric cancer progression. EBioMedicine.

[44] Zhang ZX, Zhang WN, Sun YY, Li YH, Xu ZM, Fu WN (2018). CREB promotes laryngeal cancer cell migration via MYCT1/NAT10 axis. Onco Targets Ther.

[45] Ding J, Zhang ZM, Xia Y, Liao GQ, Pan Y, Liu S, Zhang Y, Yan ZS (2013). LSD1-mediated epigenetic modification contributes to proliferation and metastasis of colon cancer. Br J Cancer.

[46] Reimer D, Hubalek M, Kiefel H, Riedle S, Skvortsov S, Erdel M, Hofstetter G, Concin N, Fiegl H, Muller-Holzner E (2011). Regulation of transcription factor E2F3a and its clinical relevance in ovarian cancer. Oncogene.

[47] Singh R, Shankar BS, Sainis KB (2014). TGF-beta1-ROS-ATM-CREB signaling axis in macrophage mediated migration of human breast cancer MCF7 cells. Cell Signal.

[48] Wierstra I, Alves J (2007). FOXM1, a typical proliferation-associated transcription factor. Biol Chem.

[49] Gu Y, Wang W, Wang X, Xie H, Ye X, Shu P (2019). Integrated network analysis identifies hsa-miR-4756-3p as a regulator of FOXM1 in triple negative breast cancer. Sci Rep.

[50] Song BN, Chu IS (2018). A gene expression signature of FOXM1 predicts the prognosis of hepatocellular carcinoma. Exp Mol Med.

[51] Caldwell SA, Jackson SR, Shahriari KS, Lynch TP, Sethi G, Walker S, Vosseller K, Reginato MJ (2010). Nutrient sensor O-GlcNAc transferase regulates breast cancer tumorigenesis through targeting of the oncogenic transcription factor FoxM1. Oncogene.

[52] Vishnubalaji R, Hamam R, Yue S, Al-Obeed O, Kassem M, Liu FF, Aldahmash A, Alajez NM (2016). MicroRNA-320 suppresses colorectal cancer by targeting SOX4, FOXM1, and FOXQ1. Oncotarget.

[53] Jiang M, Yan Y, Yang K, Liu Z, Qi J, Zhou H, Qian N, Zhou Q, Wang T, Xu X (2019). Small molecule nAS-E targeting cAMP response element binding protein (CREB) and CREB-binding protein interaction inhibits breast cancer bone metastasis. J Cell Mol Med.

[54] Paplomata E, O'Regan R (2014). The PI3K/AKT/mTOR pathway in breast cancer: targets, trials and biomarkers. Ther Adv Med Oncol.

[55] Chekhun SV, Lukyanova NY, Shvets YV, Burlaka AP, Buchinska LG (2014). Significance of ferritin expression in formation of malignant phenotype of human breast cancer cells. Exp Oncol.

[56] Park MS, Dong SM, Kim BR, Seo SH, Kang S, Lee EJ, Lee SH, Rho SB (2014). Thioridazine inhibits angiogenesis and tumor growth by targeting the VEGFR-2/PI3K/mTOR pathway in ovarian cancer xenografts. Oncotarget.

[57] Liu L, Jung SN, Oh C, Lee K, Won HR, Chang JW, Kim JM, Koo BS (2019). LAMB3 is associated with disease progression and cisplatin cytotoxic sensitivity in head and neck squamous cell carcinoma. Eur J Surg Oncol.

[58] Wang Y, Jin Y, Bhandari A, Yao Z, Yang F, Pan Y, Zheng Z, Lv S, Wang O (2018). Upregulated LAMB3 increases proliferation and metastasis in thyroid cancer. Onco Targets Ther.

[59] Ol PC, Penny SA, Dolan RT, Kelly CM, Madden SF, Rexhepaj E, Brennan DJ, McCann AH, Ponten F, Uhlen M (2013). Systematic antibody generation and validation via tissue microarray technology leading to identification of a novel protein prognostic panel in breast cancer. BMC Cancer.

[60] Beckedorff FC, Amaral MS, Deocesano-Pereira C, Verjovski-Almeida S (2013). Long non-coding RNAs and their implications in cancer epigenetics. Biosci Rep.

[61] Mercer TR, Dinger ME, Mattick JS (2009). Long non-coding RNAs: insights into functions. Nat Rev Genet.

[62] Vishnubalaji R, Shaath H, Elkord E, Alajez NM (2019). Long non-coding RNA (lncRNA) transcriptional landscape in breast cancer identifies LINC01614 as non-favorable prognostic biomarker regulated by TGFbeta and focal adhesion kinase (FAK) signaling. Cell Death Discov.

[63] Shin VY, Chen J, Cheuk IW, Siu MT, Ho CW, Wang X, Jin H, Kwong A (2019). Long non-coding RNA NEAT1 confers oncogenic role in triple-negative breast cancer through modulating chemoresistance and cancer stemness. Cell Death Dis.

[64] Fan H, Yuan J, Li X, Ma Y, Wang X, Xu B, Li X (2020). LncRNA LINC00173 enhances triple-negative breast cancer progression by suppressing miR-490–3p expression. Biomed Pharmacother.

[65] Han C, Fu Y, Zeng N, Yin J, Li Q (2020). LncRNA FAM83H-AS1 promotes triple-negative breast cancer progression by regulating the miR-136-5p/metadherin axis. Aging (Albany NY).

[66] Hua K, Deng X, Hu J, Ji C, Yu Y, Li J, Wang X, Fang L (2020). Long noncoding RNA HOST2, working as a competitive endogenous RNA, promotes STAT3-mediated cell proliferation and migration via decoying of let-7b in triple-negative breast cancer. J Exp Clin Cancer Res.

[67] Liu H, Li J, Koirala P, Ding X, Chen B, Wang Y, Wang Z, Wang C, Zhang X, Mo YY (2016). Long non-coding RNAs as prognostic markers in human breast cancer. Oncotarget.

[68] Liu Y, Sharma S, Watabe K (2015). Roles of lncRNA in breast cancer. Front Biosci (Schol Ed).

[69] Diaz-Lagares A, Crujeiras AB, Lopez-Serra P, Soler M, Setien F, Goyal A, Sandoval J, Hashimoto Y, Martinez-Cardus A, Gomez A (2016). Epigenetic inactivation of the p53-induced long noncoding RNA TP53 target 1 in human cancer. Proc Natl Acad Sci USA.

[70] Wu W, Gao H, Li X, Zhu Y, Peng S, Yu J, Zhan G, Wang J, Liu N, Guo X (2019). LncRNA TPT1-AS1 promotes tumorigenesis and metastasis in epithelial ovarian cancer by inducing TPT1 expression. Cancer Sci.

[71] Zhang Y, Sun J, Qi Y, Wang Y, Ding Y, Wang K, Zhou Q, Wang J, Ma F, Zhang J (2020). Long non-coding RNA TPT1-AS1 promotes angiogenesis and metastasis of colorectal cancer through TPT1-AS1/NF90/VEGFA signaling pathway. Aging (Albany NY).

[72] Hu C, Fang K, Zhang X, Guo Z, Li L (2020). Dyregulation of the lncRNA TPT1-AS1 positively regulates QKI expression and predicts a poor prognosis for patients with breast cancer. Pathol Res Pract.

[73] Shaath H, Vishnubalaji R, Elango R, Khattak S, Alajez NM (2021). Single-cell long noncoding RNA (lncRNA) transcriptome implicates MALAT1 in triple-negative breast cancer (TNBC) resistance to neoadjuvant chemotherapy. Cell death Discov.

[74] Zhang HM, Yang FQ, Chen SJ, Che J, Zheng JH (2015). Upregulation of long non-coding RNA MALAT1 correlates with tumor progression and poor prognosis in clear cell renal cell carcinoma. Tumour Biol.

[75] Luan W, Li L, Shi Y, Bu X, Xia Y, Wang J, Djangmah HS, Liu X, You Y, Xu B (2016). Long non-coding RNA MALAT1 acts as a competing endogenous RNA to promote malignant melanoma growth and metastasis by sponging miR-22. Oncotarget.

[76] Wu XS, Wang XA, Wu WG, Hu YP, Li ML, Ding Q, Weng H, Shu YJ, Liu TY, Jiang L (2014). MALAT1 promotes the proliferation and metastasis of gallbladder cancer cells by activating the ERK/MAPK pathway. Cancer Biol Ther.

[77] Zuo Y, Li Y, Zhou Z, Ma M, Fu K (2017). Long non-coding RNA MALAT1 promotes proliferation and invasion via targeting miR-129-5p in triple-negative breast cancer. Biomed Pharmacother.

