# Supplementary material for: Transcriptional alterations of protein coding and noncoding RNAs in triple negative breast cancer in response to DNA methyltransferases inhibition
Source: Cancer Cell Int. 2021 Sep 26;21:515. doi: 10.1186/s12935-021-02213-2 (PMC8474815; doi:10.1186/s12935-021-02213-2)
Supplement: Supplementary file 1 — Additional file 1: Figure S1. 5-mC-specific dot blot assay of gDNA of DNMT inhibitors treated TNBC models. [file 12935_2021_2213_MOESM1_ESM.pdf]

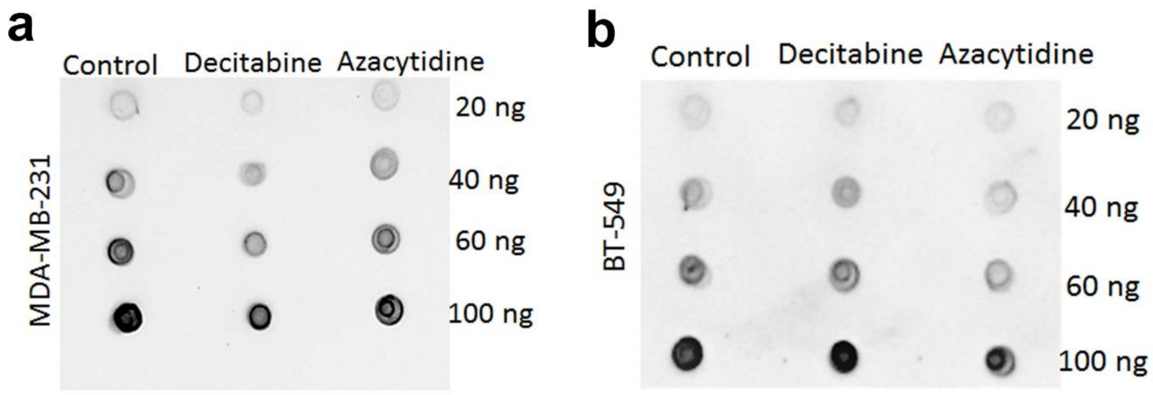

Supplementary figure 1. 5-mC-specific dot blot assay of gDNA of DNMT inhibitors treated TNBC models. Genomic DNA was isolated from control and DNMT inhibitors treated TNBC models. Different concentrations (20ng, 40ng, 60ng and 100ng) of gDNA were loaded per dot. (a) MDA-MB-231 and (b) BT-549 illustrate the images of 5-mC-specific dot blot.
